# Laparoscopic nephrectomy for giant staghorn calculus with non-functioning kidneys: Is associated unsuspected urothelial carcinoma responsible for conversion? Report of 2 cases

**DOI:** 10.1186/1471-2490-6-1

**Published:** 2006-01-08

**Authors:** Hemendra Navinchandra Shah, Pritesh Jain, Percy Jal Chibber

**Affiliations:** 1 Department of Urology, R. G. Stone Urological Research Institute, Mumbai, India; 2 Department of Urology, Grant Medical College & Sir J. J. Hospital, Mumbai, India

## Abstract

**Background-:**

Neglected renal stones remain a major cause of morbidity in developing countries. They not only result in functional impairment of affected kidney, but also act as an important predisposing factor for development of urothelial neoplasms. It is not uncommon to miss an associated urothelial tumor in a patient of nephrolithiasis preoperatively.

**Case presentation-:**

In last 3 years, we came across two patients with giant staghorn calculus and poorly functioning kidneys who underwent laparoscopic nephrectomy. In view of significant perirenal adhesions & loss of normal tissue planes both these patients were electively converted to open surgery. The pathological examination of specimen revealed an unsuspected urothelial carcinoma in both these patients. The summary of our cases and review of literature is presented.

**Conclusion-:**

It is important to keep a differential diagnosis of associated urothelial malignancy in mind in patient presenting with long standing renal calculi. The exact role of a computerized tomography and cytology in preoperative workup for detection of possible associated malignancy in such condition is yet to be defined. Similarly if laparoscopic dissection appears difficult during nephrectomy for a renal calculus with non-functional kidney, keeping a possibility of associated urothelial malignancy in mind it is advisable to dissect in a plane outside gerotas fascia as for radical nephrectomy.

## Background

Since the first clinical report in 1991, laparoscopic nephrectomy has been embraced by urologists worldwide [1]. At many medical centers, including ours, laparoscopic nephrectomy has replaced open nephrectomy as a treatment of choice for many benign and malignant diseases with excellent results [2]. From January 2000 to December 2003, 28 patients underwent laparoscopic simple nephrectomy for calculus disease with poorly functioning kidneys. Elective conversion to open surgery was needed in two patients due to presence of significant perirenal adhesions & loss of normal tissue planes. Both these patients had large staghorn calculi with a palpable kidney preoperatively. On pathological examination, the specimens revealed keratinizing type of squamous cell carcinoma & high grade transitional cell carcinoma respectively.

## Case presentations

### Case 1

**Figure 1:**
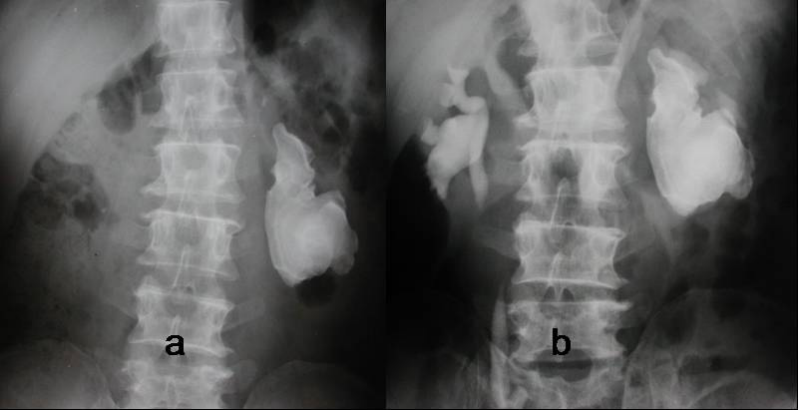
a) Plain x-ray KUB showing a large left staghorn calculus. b) Intravenous urogram showing non-visualized left kidney & normally functioning right kidney.

A 52 year old man presented for evaluation of left flank pain of 1 year duration. He was nonsmoker with a history of left nephrolithotomy for staghorn calculus 20 years back. He had hypertension and ischemic heart disease as co-morbid conditions. He also had history of pulmonary kochs in childhood. On clinical examination patient had a scar of left flank incision with a palpable hard kidney. His urine analysis showed microscopic hematuria and culture was sterile. His renal biochemistry was normal. On renal ultrasound a staghorn calculus was detected without associated hydronephrosis. The kidney was nonvisualised on intravenous urogram (figure 1) &contributed 8% of total renal function on DTPA renal scan. Patient underwent transperitoneal laparoscopic nephrectomy. During surgery there were dense perirenal adhesions especially near the hilum. During attempted adhesiolysis there was a rent in renal pelvis with resultant spillage of putty material. The hilar lymph nodes were also enlarged and densely adherent to the renal vessels. In view of past history of pulmonary kochs we suspected associated renal kochs in the patient. The procedure was electively converted to open surgery via flank incision. The total operating time was 5 hours and 20 minutes with estimated blood loss of 200 ml. The patient had a smooth post-operative recovery. The histopathological examination of the specimen revealed presence of keratinizing type of squamous cell carcinoma of renal pelvis with metastasis to hilar lymph nodes. Patient was advised postoperative palliative chemo-radiotherapy. After six weeks of surgery patient developed fulminant herpes zoster and expired due to same.

### Case 2

**Figure 2:**
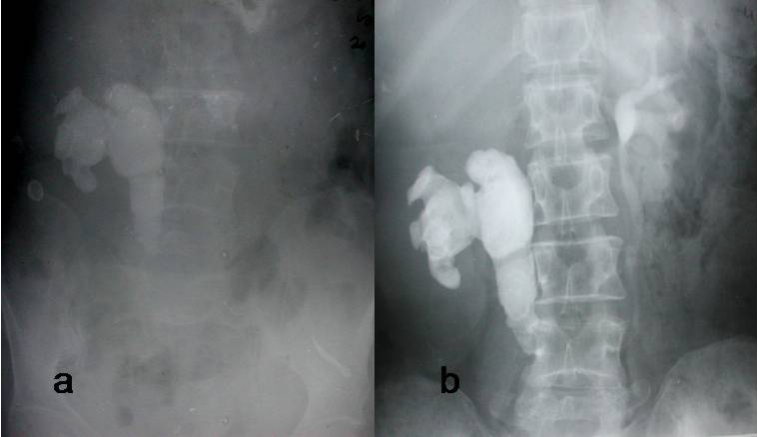
a) Plain x-ray KUB revealing a large right staghorn calculus extending in the upper ureter. b) Intravenous urography showing non-visualized right kidney with normally functioning left kidney.

A 54 year old female presented with complains of right flank pain and dysuria of 2 year duration. There was no significant past history & on clinical examination she had a palpable right kidney. She was anemic with preoperative hemoglobin of 9.2 gm%. Her renal biochemistry was normal. On sonography she had a large renal & upper ureteric calculus with mild hydronephrosis. On intravenous urography right kidney was not visualized while the left kidney showed normal function (figure 2). The differential function on DTPA renal scan revealed that right kidney contributed 12 % to the total renal function. In view of patient's age, normal serum creatinine & normally functioning opposite kidney, option of renal conservation versus nephrectomy were discussed with patient. She opted for retroperitoneoscopic nephrectomy. Intraoperatively there were dense adhesions of kidney with duodenum & inferior venacava with loss of normal tissue planes. To continue laparoscopic dissection was considered hazardous & the procedure was electively converted to open surgery through flank approach. Nephrectomy with removal of ureter upto pelvic brim was done to remove the stone bearing part of ureter after extending the incision anteriorly in paramedian fashion. The total operating time was 4 hours and 45 minutes with estimated blood loss of 700 ml. Patient was transfused two units of blood postoperatively. The intraoperative findings raised a suspicion of xanthogranulomatous pyelonephritis. However the  histopathological examination of specimen revealed  high grade transitional cell carcinoma of renal pelvis  with infiltration of renal parenchyma and ureteric  adventitia. The renal hilar lymph nodes were not involved with disease. The cut margin of ureter revealed carcinoma insitu. Patient was informed about need for segmental ureterectomy and lymphadenectomy. However she was not willing for any surgical intervention. She was treated with combination chemotherapy. On follow-up at 6 months she had developed massive pulmonary metastasis.

## Conclusion

Long standing calculus disease is known to cause functional impairment of kidney. The pathological processes identified in a poorly functioning kidney secondary to calculus disease include parenchymal atrophy, chronic pyelonephritis, xanthogranulomatous pyelonephritis & rarely associated urothelial carcinoma. Li & Cheung et al found that 2% of their patients with recurrent staghorn stones had squamous cell carcinoma of renal pelvis [3]. In their  review of literature they noted that urothelial  malignancy should be suspected in patients with a long  standing history of stones, pain, urinary tract  infection and a palpable kidney, especially when the  enlargement is out of proportion to the hydronephrosis  shown on the intravenous urography &/or ultrasonography. Radiological investigations like intravenous urography, ultrasound or computed tomography are not diagnostic [4]. Reactive fibrosis and postobstructive inflammatory changes can mimic tumor invasion on computed tomography, and in such cases staging will not be accurate [5]. Recently sonographic finding of markedly enlarged and  hydronephrotic kidney with obstructive calculi,  stigmata of pyonephrosis and, anechoic pelvic mass,  extending in calyces is found to help in preoperative  diagnosis of associated urothelial malignancy in  patients with renal calculi [6,7]. Similarly urinary calculi are likely to produce a variety of unusual alterations in the morphology of urothelial cells, making urinary cytology less reliable to diagnose coexistent urothelial carcinoma [8]. Hence it is not uncommon to miss an associated urothelial tumor in a patient of nephrolithiasis preoperatively. Ozdamar AS et al propose to take biopsies of the urothelium in every stone surgery & if any suspicious finding is demonstrated, the patients should be enrolled in a follow-up programme or should be transferred to tumor treatment programme [9].

Umbas  Rainy described a series of 4 patients with renal  squamous cell carcinoma, three of which were detected  postoperatively after nephrectomy for non-functioning  kidney [8]. Similarly there are multiple case reports of urothelial cancers associated with renal stone disease or hydronephrosis [10-13]. In both of our cases the tumor was detected postoperatively during histopathological examination of nephrectomized specimen. An elective conversion to open surgery was required in both patients in view of significant perirenal adhesions especially near hilum.

We started laparoscopic nephrectomy in our department from January 2000. Initially we employed transperitoneal approach and later on with increasing experience we progressed towards retroperitoneoscopy. Hence first case in our report was approached transperitoneally and the second case retroperitoneoscopically. In our cases the difficulty encountered during laparoscopic dissection was probably due to associated locally advanced urothelial carcinoma. An elective conversion during laparoscopic nephrectomy is described for kidney with associated renal tuberculosis, pyonephrosis & chronic pyelonephritis especially xanthogranulomatous pyelonephritis. An extensive Medline search failed to reveal unsuspected urothelial carcinoma in a non-functioning kidney due to calculus disease diagnosed after a conversion during laparoscopic nephrectomy. Retrospectively, we noted that both of our patients had palpable kidneys, which were out of proportion to hydronephrosis seen on ultrasound. It is important to keep a differential diagnosis of associated urothelial malignancy in mind in patient presenting with long standing renal calculi. We had not done computerized tomography in our patients, and retrospectively feel that in first patient we could have identified lymphadenopathy on the computerized tomography. Although the exact role of a computerized tomography and cytology in preoperative workup for detection of possible associated malignancy in such condition is yet to be defined, computerized tomography may help in identifying xanthogranulomatous pyelonephritis or renal mass with or without associated lymphadenopathy in such scenario. Similarly if laparoscopic dissection appears difficult during nephrectomy for a renal calculus with non-functional kidney, keeping a possibility of associated urothelial malignancy in mind it is advisable to dissect in a plane outside gerotas fascia as for radical nephrectomy. Similarly the kidneys should be removed via laparoscopic bag when the procedure is completed laparoscopically. Tan YH et al compared outcome of 22 patients who underwent hand-assisted laparoscopic nephrectomy for inflammatory renal conditions with those patients undergoing surgery for renal tumors [14]. They found that the patients with inflammatory pathology had longer mean operative times, higher estimated blood loss, longer hospital stay, and higher morbidity than patients undergoing radical nephrectomy. In cases of difficult dissection during simple nephrectomy a sound judgement of conversion to open surgery is essential. As concluded by Shekarriz B et al, higher conversion rate and longer operative time should be expected in such patients. Early conversion may be required due to failure to progress [15].

## Competing interests

The author(s) declare that they have no competing interests.

## Pre-publication history

The pre-publication history for this paper can be accessed here:



## References

[B1] Clayman RV, Kavoussi LR, Soper NJ, Dierks SM, Meretyk S, Darcy MD, Roemer FD, Pingleton ED, Thomson PG, Long SR (1991). Laparoscopic Nephrectomy: Initial case report. J Urol.

[B2] Gill IS, Kavoussi LR, Clayman RV, Ehrlich R, Evans R, Fuchs G, Gersham A, Hulbert JC, McDougall EM, Rosenthal T, Schuessler WW, Shepard T (1995). Complications of laparoscopic nephrectomy in 185 patients: A multiinstitutional review. J Urol.

[B3] Li MK, Cheung WL (1987). Squamous cell carcinoma of the renal pelvis. J Urol.

[B4] Blacher EJ, Johnson DE, Abdual-Karim FW, Ayala AG (1985). Squamous cell carcinoma of renal pelvis. Urology.

[B5] Cholankeril JV, Freundlich R, Ketyer S, Spirito AL, Napolitano J (1986). Computed tomography in urothelial tumors of renal pelvis and related filling defects. J Comput Tomogr.

[B6] Tlili-Graiess K, Gharbi-Jemni H, Atallah R, Krid M, Abbassi-Bakir D, Allegue M, Kraiem C, Mosbah F, Jeddi M (1994). Ultrasonographic aspects of kidney pelvis tumors associated with infectious hydronephrosis of lithiasic origin. J Radiol.

[B7] Jemni M, Mosbah A, Allegue M, Bouzakoura C, Hamida C (1987). Renal lithiasis and urothelial tumor. Ann Urol (Paris).

[B8] Sangisetty KV, Randrup ER (1985). Congenital giant hydronephrosis with unsuspected transitional cell carcinoma. Urology.

[B9] Ozdamar AS, Ozkurkcugil C, Gultekin Y, Gokalp A (1997). Should we get routine urothelial biopsies in every stone surgery?. Int Urol Nephrol.

[B10] Sheaff M, Fociani P, Badenoch D, Baithun S (1996). Verrucous carcinoma of the renal pelvis: case presentation and review of the literature. Virchows Arch.

[B11] Fekak H, Rabii R, Moufid K, Joual A, Dahami Z, Mrini M (2002). Unusual clinical presentations of tumours of the renal pelvis. Report of two cases. Prog Urol.

[B12] Medina Perez M, Valpuesta Fernandez I, Valero Puerta J (1998). Pyelocaliceal urothelial carcinoma associated with pelvis lithiasis. Arch Esp Urol.

[B13] Ueno M, Jitsukawa S, Nakashima J, Ban S, Deguchi N (2002). Primary transitional cell carcinoma insitu and renal calculus in the dilated pelvis. BJU International.

[B14] Tan YH, Siddiqui K, Preminger GM, Albala DM (2004). Hand-assisted laparoscopic nephrectomy for inflammatory renal conditions. J Endourol.

[B15] Shekarriz B, Meng MV, Lu HF, Yamada H, Duh QY, Stoller ML (2001). Laparoscopic nephrectomy for inflammatory renal conditions. J Urol.

